# Supplementary material for: Senescent Macrophages Release Inflammatory Cytokines and RNA-Loaded Extracellular Vesicles to Circumvent Fibroblast Senescence
Source: Biomedicines. 2024 May 14;12(5):1089. doi: 10.3390/biomedicines12051089 (PMC11118806; doi:10.3390/biomedicines12051089)
Supplement: Supplementary file 1 [file biomedicines-12-01089-s001.zip › biomedicines-2974378-supplementary.pdf]

biomedicines

MDPI

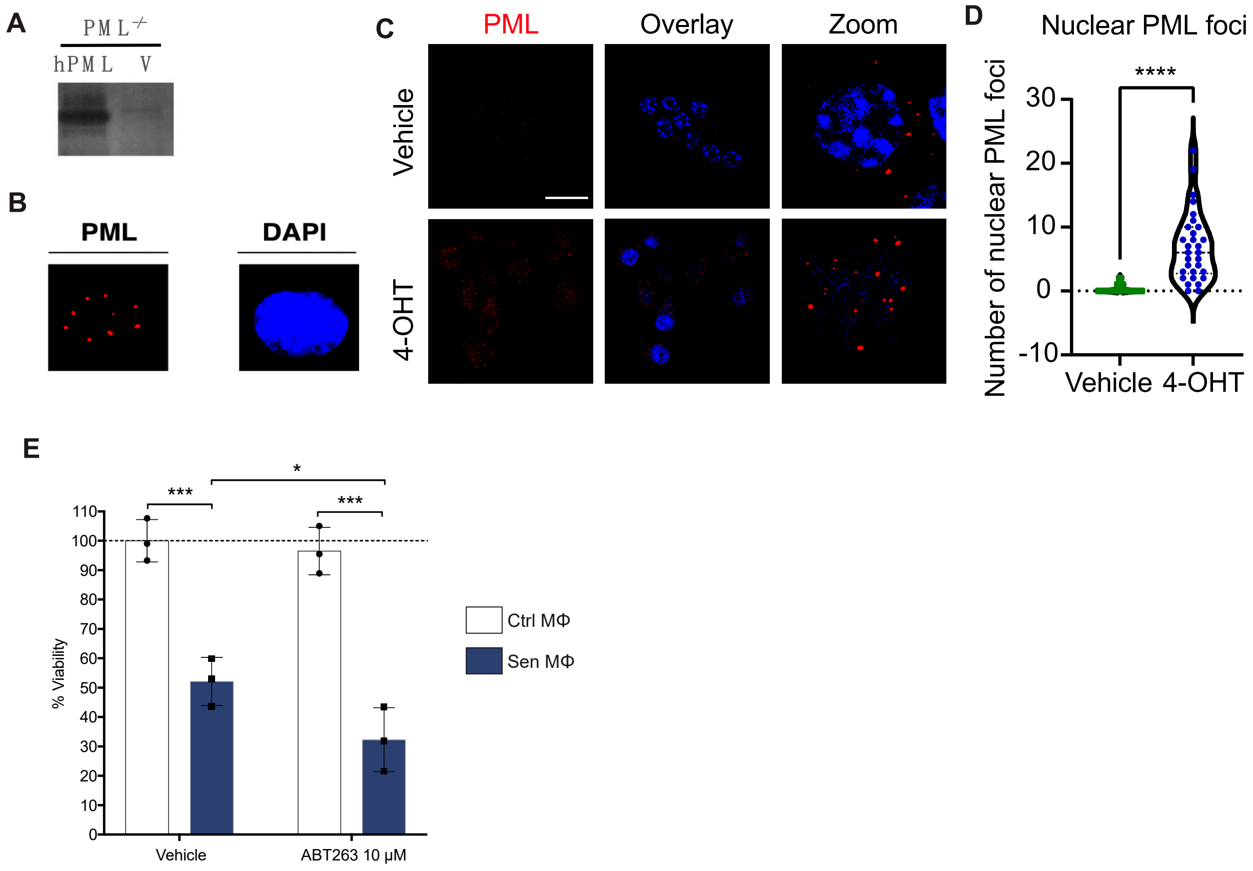

Figure S1. Additional senescent traits in RAW 264.7 murine macrophages expressing activated Raf. A-B) Development of antibodies against PML. To obtain an anti-PML antibody we synthesized a peptide corresponding to aminoacids 352 to 366 of human PML: LRQALCRLRQEEPQS. The corresponding mouse region is LRKALCSLRQEEPQN (changes are underlined). This sequence is present in all known PML isoforms. In western blot, the polyclonal antibody recognized PML in Pml null MEFs expressing or not human PML (A). In immunofluorescence (IF) the antibody recognized the increase of PML bodies after senescence induction using oncogenic Ras in human fibroblasts (B). C) IF staining of nuclear PML foci and DAPI staining performed on RAW 264.7 cells expressing ΔRaf-1:ER and treated with Vehicle or 4-OHT (100nM, 3 days). D) Quantification of IF from (C) showing the number of nuclear PML foci per cell. The experiments were performed 3 times. **** p-value < 0.0001 using Mann-Whitney U Test. E) Viability of Ctrl MΦ and Sen MΦ following treatment with the senolytic 10 μM of Navitoclax (ABT-263) or vehicle. Viability was determined by Crystal Violet retention assay. The experiment was performed 3 times. Error bars represent standard deviation. * p-value ≤ 0.05 ; *** p-value < 0.001 using ANOVA.

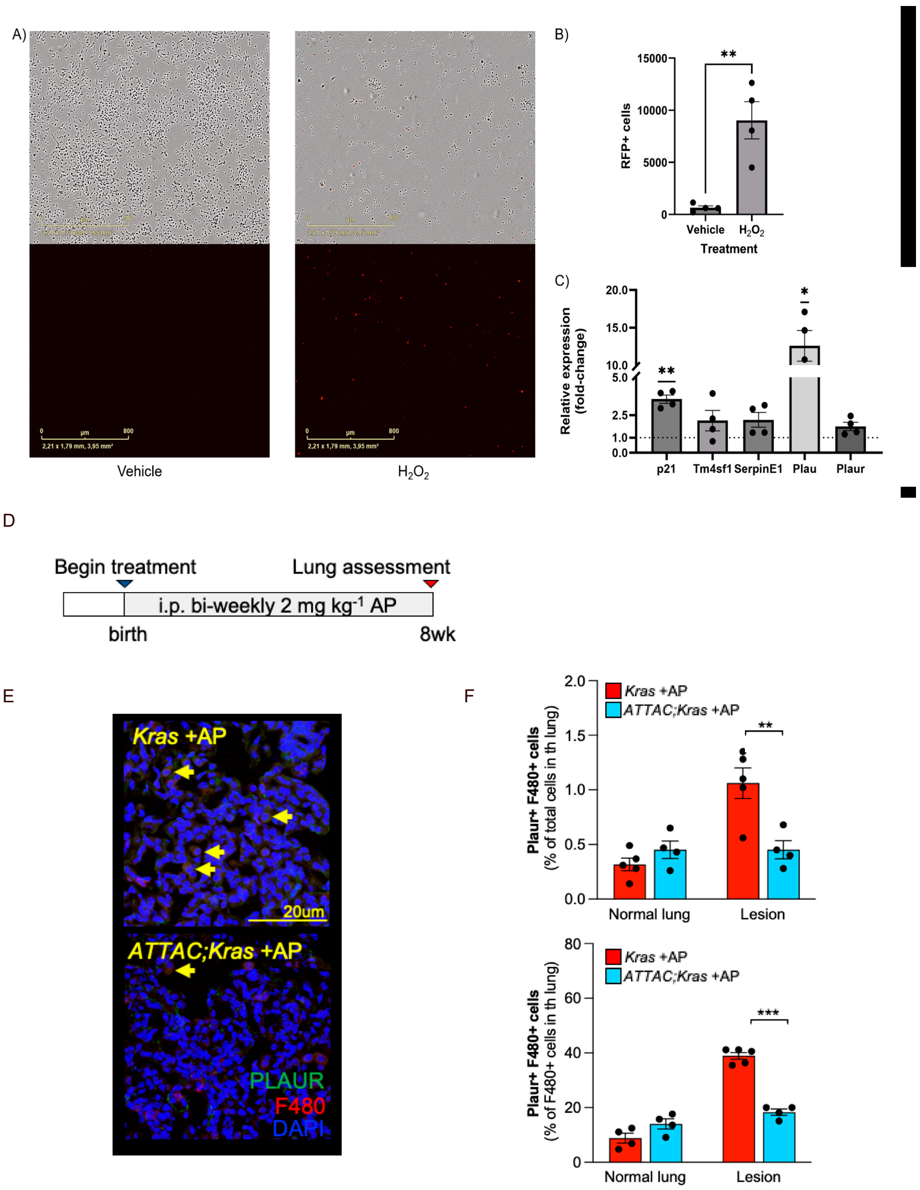

Figure S2. Other models of macrophage senescence.

A) Induction of senescence in mouse BMDM. BMDM isolated from P16-3MR mice were treated for 2 hours with 500 µM H_2O_2 at day 3 of differentiation and senescence markers were assessed on day 6. A) IncuCyte photos of both phase and red fluorescence (top) and only red fluorescence (bottom) (A) and IncuCyte analysis of the number of RFP+ cells in wells (B) indicate an increase in the RFP senescence marker. C) qPCR for the expression of senescence and SASP-associated genes. * : P<0.05, ** : P<0.01. N=4. D) Experimental design for in vivo removal of p16INK4a positive cells in *INK-ATTAC; Kras* mice. E-F) Representative immunofluorescence image (E) and quantification (F) of Plaur+; F4/80+ cells. relative to the % of total cells in the lung or the % of F4/80+ cells in the Kras or in the *INK-ATTAC; Kras* mice model control or treated with AP (AP20187) as illustrated in D. Data are means ± SEM. *$p < 0.05$; **$p < 0.01$; ***$p < 0.001$; unpaired two-tailed Student's t test.

A

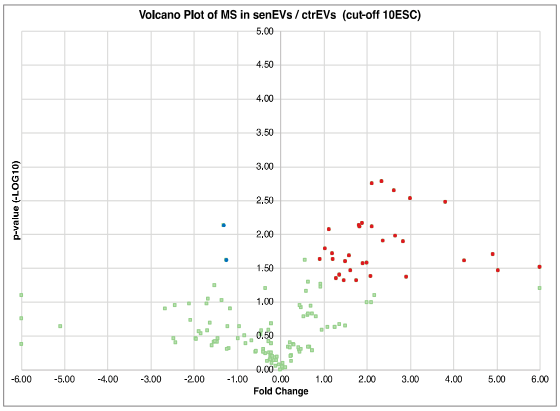

B

>= 10ESC >=1.5fold <=p0.05	fold change pos/neg log2	p value (of t test)
Aldoa	1.4695	0.04863
Anpep	2.8440	0.01286
Basp1	3.8074	0.00334
Ca2	2.6323	0.00226
Cap1	1.3576	0.03999
Clic1	1.1155	0.00851
Ehd4	1.8995	0.02724
Esd	1.5850	0.02078
Hmga2	1.6215	0.03486
Lamp1	3.0000	0.00300
Lpl	-1.3219	0.00748
Msn	2.3458	0.00169
Mvp	6	0.03079
Myh9	1.8249	0.00789
Pcna	-1.2515	0.02417
Pdcd6ip	2.0809	0.04195
Plau	5.0297	0.03498
Prdx1	1.2044	0.01933
Rab5c	4.2479	0.02483
Rab8b	2.1155	0.00780
Rpl18	1.2065	0.02354
Rpl3	4.9069	0.02011
Rpl4	1.7590	0.04907
Rpl6	2.0000	0.02638
Rplp0	0.9151	0.02370
Rplp2	1.0385	0.01638
Rps9	2.3656	0.01259
Sdcbp	1.5025	0.02549
Snx9	2.9069	0.04254
Srgn	1.2824	0.04517
Tln1	1.8845	0.00698

C

D

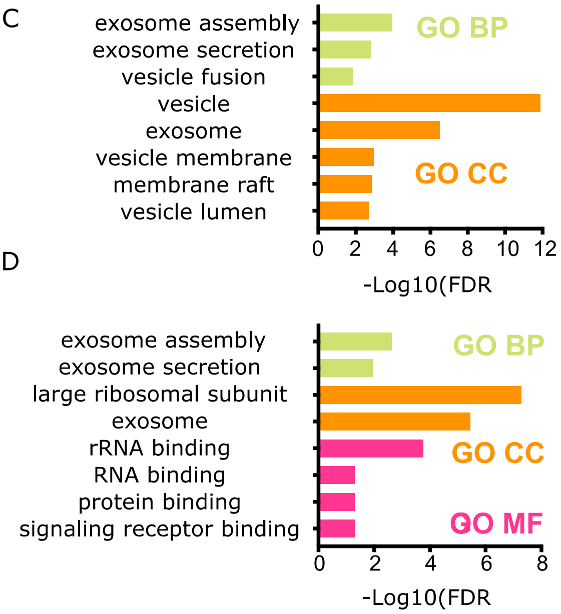

Figure S3. Proteomics of senescent EVs.
A) Volcano plot of proteins identified in the Mass Spectrometry (MS) analysis of proteins in EVs isolated from the conditioned media of senescent RAW 264.7 RAF-ER cells after 3 days OHT stimulation (senEVs) or control cells (3 days vehicle, ctrEVs). Analysis was conducted with three biological replicates and only proteins with at least 10 exclusive spectral counts (ESCs) were kept to evaluate the fold change (mean number of peptides from senEVs / ctrEVs) and p-value. Green: all proteins. Blue: proteins significantly more present in ctrEVs. Red: proteins significantly more present in senEVs. We set significance at 1.5-fold or greater with a p-value of 0.05 or smaller. B) List of the proteins significatively regulated in senEVs vs ctrEVs with their fold change (log2) and p-value. C) GO Term analysis of all 124 proteins identified by Mass Spectrometry (MS) in EVs derived from Ctrl and Sen MΦ. MS data was analyzed with Exclusive spectrum counts (ESC) ≥ 10. BP: Biological Process, CC: Cellular component. D) GO Term analysis of the 31 proteins from (B). BP: Biological Process, CC: Cellular component, MF: Molecular function.

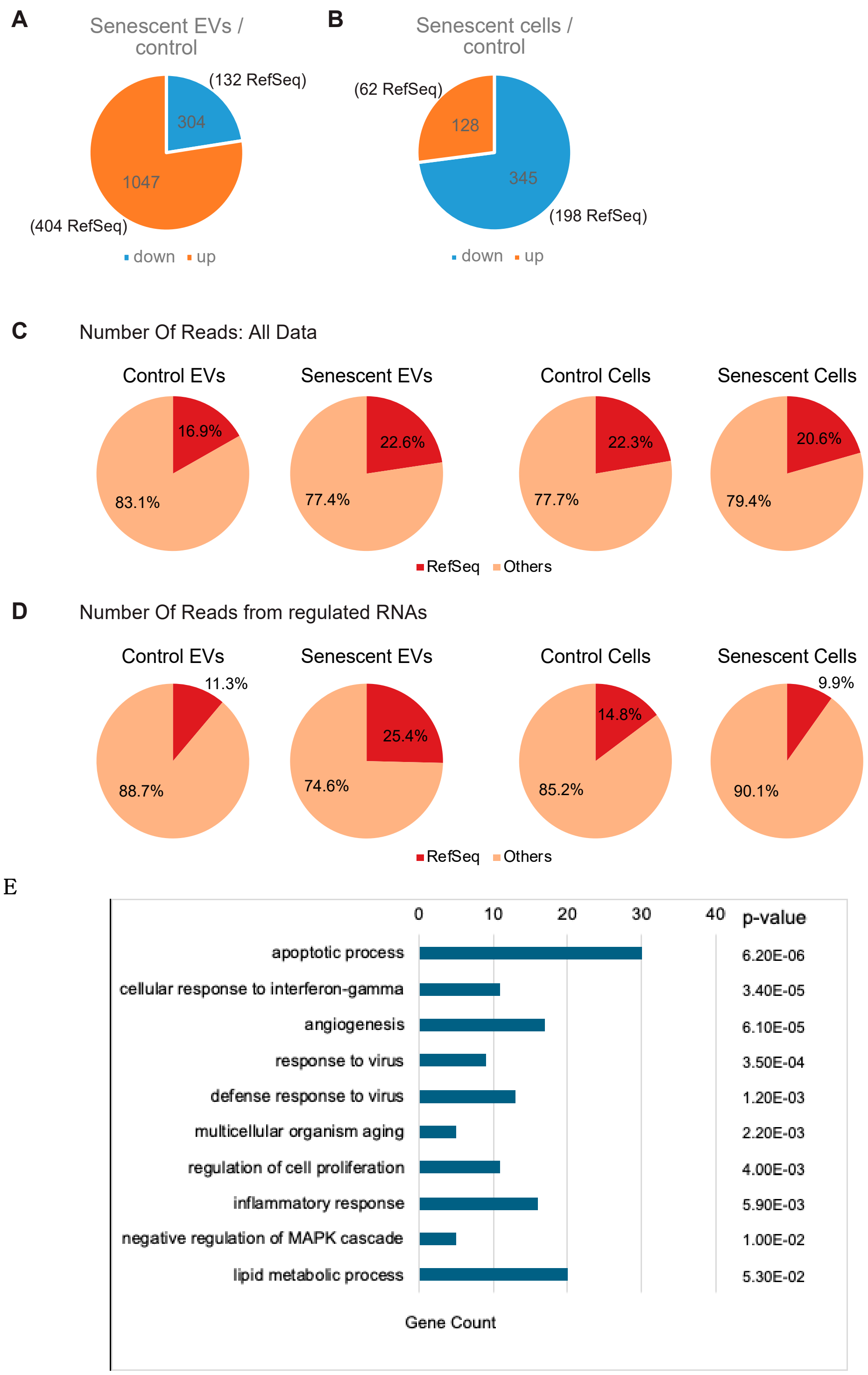

Figure S4. Changes in mRNA cargo in EVs and in macrophages upon senescence.
A-B) Pie charts of RNAs upregulated (UP) and downregulated (DOWN) identified by small RNAseq in in EVs from Sen MΦ vs Ctrl MΦ (A) or in Sen MΦ vs Ctrl MΦ (B). Fold Change ≥ |2| and p-adjusted ≤ 0.005. The number of RNA species (i.e.: tRNAs, mRNAs, rRNA and all others) are within the circles while the number of mRNAs only (RefSeq) is indicated outside the circle. C) Relative proportion of reads from mRNAs vs. other RNAs in control EVs, senescent EVs or their cellular sources. D) Relative proportion of reads from regulated mRNAs vs. other RNAs in control EVs, senescent EVs or their cellular sources. E) Gene Ontology (GO) enrichment analysis of mRNAs found upregulated in senescent EVs in comparison with control EVs.
